# A Rare Infection Post Hyaluronic Acid Injection in the Trochanter: Case Report and Literature Review

**DOI:** 10.1093/asjof/ojae052

**Published:** 2024-06-28

**Authors:** Mohamed Badie Ahmed, Fatima Saoud Al-Mohannadi, Amina Bensaoua, Mansour Binfayed, Abeer Alsherawi

## Abstract

Filler injection has become a commonplace aesthetic procedure. Although the incidence of infection following filler injections is typically low, ranging between 0.04% and 0.2%, the potential consequences can be serious. In this manuscript, we present the case of a 29-year-old female patient who presented to our emergency department after receiving a hyaluronic acid filler injection in the trochanteric area at a private center. She developed signs and symptoms shortly after the procedure, including bilateral hip pain and a fever that persisted for 3 days. Despite initial antibiotic and pain medication treatment, the patient’s condition continued to worsen. Incision and drainage were performed, and pus culture revealed profuse Extended Spectrum Beta-Lactamase (ESBL)-producing *Serratia marcescens*. The patient experienced clinical improvement following the incision and drainage procedure and initiation of antibiotics sensitive to the pathogen. Although rare, *S. marcescens* infection following filler injections has been reported in 2 previous cases. Therefore, early recognition of infection signs and symptoms is crucial to mitigate the severity of consequences and improve outcomes. Aggressive surgical and medical interventions, such as incision and drainage, debridement, and appropriate antibiotic therapy, may be necessary to achieve a successful outcome.

**Level of Evidence: 5:**

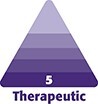

The field of aesthetic surgery and medicine has witnessed a remarkable rise in popularity over recent years, with an increasing number of individuals seeking cosmetic enhancement procedures, for example, liposuction, lipofilling, and soft-tissue fillers. Although these procedures are generally considered safe, the rising incidence of associated soft-tissue infections presents a significant cause for concern among healthcare providers and patients.^1^

One of the most performed procedures is dermal and soft-tissue filler injections, which offer quick and minimally invasive solutions for restoring volume, facial and body contouring, and rejuvenating the skin. These fillers are divided into 3 major groups: temporary, semi-permanent, and permanent. Hyaluronic acid (HA)-based fillers fall into the temporary group and are the most commonly used due to their safety and efficacy.^2^ Despite their widespread use and safety, complications can occur, including pigmentary changes, hypersensitivity reactions, infections, nodule formation, granulomatous reactions, vascular occlusion, and migration of filler material.^3^ The infection rate after filler injections is low, approximately ranging from 0.04% to 0.2%.^4^ However, it is worth noting that these infections can occur either immediately or be delayed for several years following the procedure.^5^

In this report, we present a case of post-HA filler infection with *Serratia marcescens*, occurring in an immunocompetent patient at the trochanters. Our aim is to bring attention to this uncommon, yet potentially serious complication associated with HA filler procedures. Furthermore, we emphasize the importance of promptly recognizing and managing such infections to minimize adverse sequelae and optimize patient outcomes.

This study followed the principles of the Declaration of Helsinki. Written informed consent was obtained from the patient for publication of this case report and any accompanying images.

## CASE PRESENTATION

In March 2024, a 29-year-old female patient presented to our emergency department with bilateral hip pain and fever for 3 days. One day prior to the onset of symptoms, the patient had cosmetic filler injections in the trochanteric areas bilaterally. She received 30 mL of HA filler injections on each side under sterile conditions and local anesthesia. There were no immediate adverse events except for the expected mild redness and swelling at the injection sites postprocedure. The patient was reassured and discharged on prophylactic amoxicillin–clavulanic acid 1 g twice daily along with symptomatic relief medications. On the next day, the patient started experiencing fever at night, documented at 38°C with some night sweats. There was no increase in redness or swelling. The fever continued to occur, and on the third day, the patient developed sudden-onset severe hip pain bilaterally with increasing redness and local warmth at the site of injections. Pain was characterized as sharp and shooting, aggravated by movement, with no relief with simple analgesics.

Upon examination, vitals were within normal limits except for mild tachycardia that settled after adequate pain control with a morphine injection. Local skin examination revealed bilateral swelling and redness ∼9 × 11 cm in size, with increased warmth, tenderness, and multiple indurations on palpation. Initial laboratory tests revealed mild neutrophilic leukocytosis with an elevated c-reactive protein of 150. The patient underwent ultrasound imaging of bilateral hips, which showed superficial, ill-defined hypo-hyperechoic areas with subcutaneous edema, the largest being 1.87 cc (1.5 × 1.8 × 1.2 cm) in the right hip and 15 cc (2.2 × 1.3 × 2.5 cm) in the left hip.

**Figure 1.**
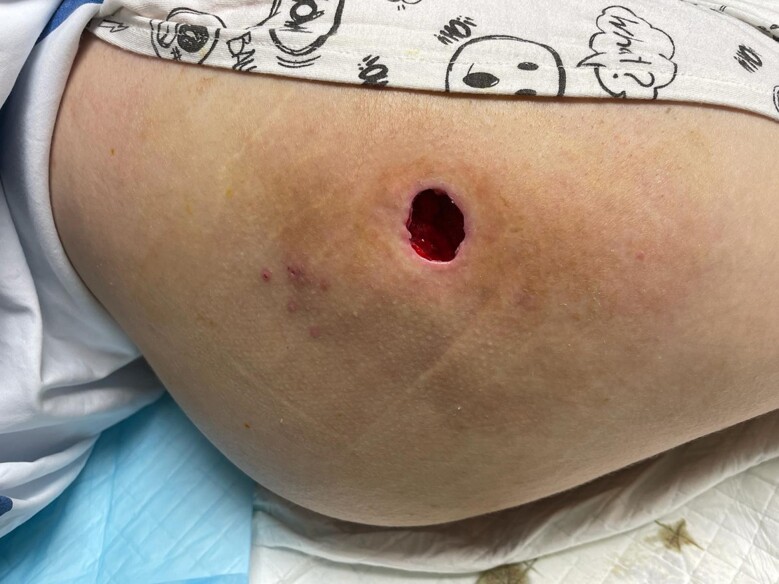
Postoperative photograph of the left trochanter showing a bigger open wound cavity compared to the right side surrounded by mild erythema, indicating resolution of the infection.

**Figure 2.**
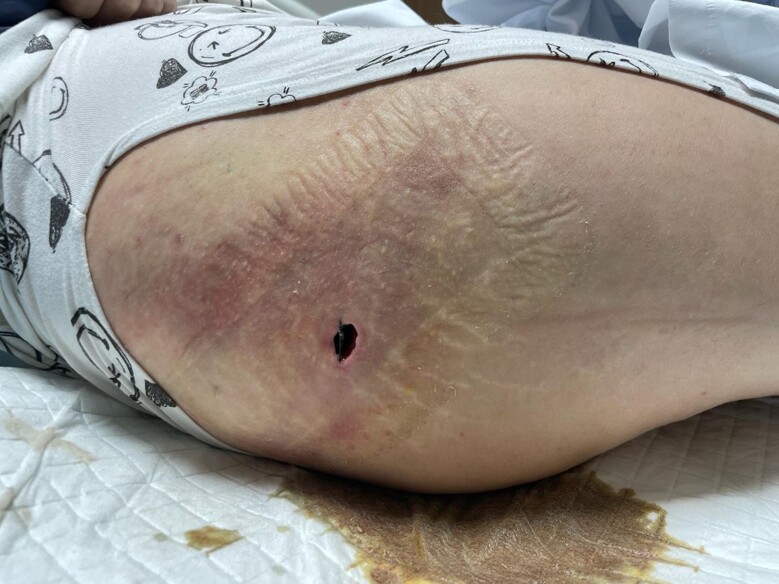
Postoperative photograph of the right trochanter showing an open wound cavity surrounded by mild erythema, indicating resolution of the infection.

The patient was admitted under the plastic surgery team. She was started on IV clindamycin along with adequate pain medications, requiring multiple doses of morphine injections without significant pain relief. After serial examinations, bedside incision and drainage were performed for pain relief, which yielded frank pus. Swabs were taken for culture and sensitivity testing. The patient was scheduled for theater incision and drainage under spinal anesthesia the next day (Figures 1, 2). Intraoperative findings revealed multiple superficial abscesses at the sites of filler injections. Approximately 60 mL of pus was drained from each side, and washout of filler injections was performed. A drain was inserted along with ribbon gauze packs.

The patient had no immediate complications. She reported improving pain postoperatively. The cultures of the tested swab grew profuse Extended Spectrum Beta-Lactamase (ESBL)-producing *S. marcescens*, resistant to ampicillin and amoxicillin–clavulanate, but sensitive to ertapenem and trimethoprim-sulfamethoxazole (TMP–SMX). The antibiotics were then changed to daily IV ertapenem with daily dressing of the wounds. A follow-up soft-tissue ultrasound showed resolution of the previous collections with only mild soft-tissue edema. Subsequently, the patient continued to show clinical improvement with inflammatory markers trending down. The patient was then discharged after confirming resolution of the wound infection and proper healing, with future follow-up scheduled in the plastic surgery outpatient department.

## DISCUSSION

The incidence of infections following filler injections is relatively rare. The occurrence of such infections depends on product type, sterility, method of injection, and patient background. In addition, it has been estimated that postdermal filler infections have a rate of only 0.04% to 0.2% of cases.^4^ Despite the low prevalence, it is crucial to recognize that these infections may manifest either immediately or with delayed onset. This delayed onset poses a challenge for both patients and healthcare providers, as it can lead to unexpected complications and necessitate long-term monitoring. Among the pathogens associated with postfiller infections, *S. marcescens*, a gram-negative bacillus, is considered rare, especially in immunocompetent patients. *Serratia marcescens* is typically known as an opportunistic pathogen that primarily causes nosocomial infections in immunocompromised individuals.^6^ However, the pathogenesis of *S. marcescens* in soft-tissue infections among immunocompetent patients following filler injections remains poorly understood, with only 2 documented cases in the medical literature post facial filler injections in immunocompetent patients.^7,8^ Nevertheless, the consequences can be severe, leading to disfigurement, tissue necrosis, necrotizing fasciitis, and systemic complications if not promptly diagnosed and treated.^9^

Our report presents a case of an immediate *S. marcescens* infection in an immunocompetent female following trochanteric HA filler injection. The product used in our case was a cross-linked HA, each 1 mL of the product contains 6.9 mg of sodium chloride, 2 mg of HA, and 20 mg of cross-linked HA. Two similar cases were reported in the literature. The first case involved a 62-year-old immunocompetent female who developed cutaneous *S. marcescens* infection of the glabella following glabella and forehead HA filler injections. However, this patient also received a glabellar area permanent filler injection containing polymethylmethacrylate 2 years prior to her presentation, and the HA injection took place 3 months prior to her presentation. Therefore, this could have served as a niche for the entry of *S. marcescens* microorganisms. Initially, topical and systemic antibiotics were tried along with multiple incisional drainages, yet the condition persisted, fluctuating in severity over time. However, after tissue and pus cultures confirmed the pathogen and sensitivity results, the patient was treated with 2 incision and drainage procedures along with oral TMP–SMX and ciprofloxacin, resulting in full resolution of the infection without recurrence after 1 year of follow-up. The other case involved a 70-year-old immunocompetent male patient with no comorbid disorders who presented with painful nodules in the nasolabial fold around 28 days after cosmetic eyelid surgery and filler injection. No information was provided regarding the type of filler injected in the area. A total of 13 cases were reported in the same paper, and 17 strains were isolated from the wounds. All of them were resistant to cefazolin, and 16 out of 17 were resistant to amoxicillin–clavulanic acid and ampicillin. Additionally, all remained susceptible to third- and fourth-generation cephalosporins, gentamicin, levofloxacin, and meropenem.

In comparison with other cases, we find our case unique, because, to our knowledge, it is the first case to report *S. marcescens* infection posttrochanteric HA filler injection. Moreover, our patient is younger (29 years) compared with the other 2 patients (62 and 70 years), highlighting that it can present in a wide age range. Furthermore, our susceptibility results showed resistance to amoxicillin–clavulanic acid and ampicillin, similar to the majority of cutaneous *S. marcescens* cases mentioned in the aforementioned study.^10,11^ Additionally, the early presentation of the case (a few days after the procedure) was a critical factor in early intervention and control of the infection. Source control was achieved by incision and drainage, and the placement of a drain after the surgery helped reduce the risk of re-collection in the cavity and expedited the healing process.^12^ This case report describes a single patient, which limits the ability to draw a broader conclusion regarding post-HA filler infections. In addition, it lacks comprehensive information about the setting of which the procedure was performed in. Therefore, it is difficult to establish at which stage the pathogen was introduced. Moreover, there is no control group to help differentiate if the postdermal filler infection was due to the technique used or the patient's health status. Although these types of infections are rare, thorough explanation and discussion with the patients, along with informed consent signing, should take place before any procedure.^13^ This will allow patients to make an informed decision before undergoing any procedure. Also, it is essential to preserve complete documentation of the consent in the patient's notes.

## CONCLUSIONS

Filler injections are very common cosmetic procedures performed worldwide, and they can be associated with many complications. Moreover, procedure performers should have a high index of suspension to detect abnormal signs and symptoms during the early days and be mindful about the technique and volume of fillers planned for patients. Rapid recognition of infection and early intervention are key factors to avoid devastating outcomes.
